# Osteochondral resurfacing implantation angle is more important than implant material stiffness

**DOI:** 10.1002/jor.24101

**Published:** 2018-07-13

**Authors:** Ashley Heuijerjans, Wouter Wilson, Keita Ito, Corrinus C. van Donkelaar

**Affiliations:** ^1^ Orthopaedic Biomechanics Department of Biomedical Engineering Eindhoven University of Technology P.O. Box 513 5600MB Eindhoven The Netherlands

**Keywords:** articular cartilage defect, collagen damage, osteochondral implant, finite element analysis, motion

## Abstract

Osteochondral resurfacing implants are a promising treatment for focal cartilage defects. Several implant‐factors may affect the clinical outcome of this treatment, such as the implant material stiffness and the accuracy of implant placement, known to be challenging. In general, softer implants are expected to be more accommodating for implant misalignment than stiffer implants, and motion is expected to increase effects from implant misalignment and stiffness. 3D finite element models of cartilage/cartilage contact were employed in which implantation angle (0°, 5°, 10°) and implant material stiffness (*E* = 5 MPa, 100 MPa, 2 GPa) were varied. A creep loading (0.6 MPa) was simulated, followed by a sliding motion. Creep loading resulted in low maximum collagen strains of 2.5% in the intact case compared to 11.7% with an empty defect. Implants mostly positively affected collagen strains, deviatoric strains, and hydrostatic pressures in the adjacent cartilage, but these effects were superior for correct alignment (0°). The main effect of implant misalignment was bulging of opposing cartilage tissue into the gap caused by the misalignment. This increased collagen strains and hydrostatic pressures. Deviatoric strains were increased adjacent to the gap. Subsequent sliding initially increased strains for a stiff, misaligned implant, but generally sliding decreased strains. In conclusion, implants can decrease the detrimental effect of defects, but correct implant alignment is crucial, more than implant material stiffness. Implant misalignment causes a gap, causing potentially damaging cartilage deformation during prolonged loading, for example, standing, even for soft implants. Mild motion may positively affect the cartilage. © 2018 The Authors. Journal of Orthopaedic Research® published by Wiley Periodicals, Inc. on behalf of Orthopaedic Research Society. J Orthop Res 36:2911–2922, 2018.

Focal cartilage defects are a common type of joint injury^1^, ^2^ which can be very painful and cause severe disability.^3^, ^4^ Such focal defects usually progress into osteoarthritis.^5^, ^6^, ^7^, ^8^ Common treatments of such defects include a marrow‐stimulating therapy called microfracturing or a cell‐based regenerative therapy called autologous chondrocyte implantation (ACI, Jeuken et al.^9^). However, both treatment strategies depend on the regenerative potential of the patient. For older patients with reduced regenerative capability, tissue adaptation will be limited to the spreading of tissue damage. For these patients, non‐resorbable resurfacing implants may be a promising alternative treatment. Osteochondral resurfacing implants have the potential to allow the patient to maintain an active lifestyle, and in addition, delay the need for a total knee arthroplasty (TKA). Metal resurfacing implants, such as HemiCap® (Arthrosurface INC., Franklin, MA) and Episealer® Condyle Solo (Episurf Medical AB, Stockholm, Sweden), are already commercially available and/or are in the process of testing in human trials.^10^, ^11^ A polymeric/metal hybrid resurfacing implant called BioPoly™ is also in clinical trial.^12^

The use of materials with different mechanical properties for resurfacing implants, such as metals^13^, ^14^ with a stiffness around 35 GPa,^15^ polymers of a wide range of stiffnesses depending on the composition, autologous cartilage, and bone in the case of mosaicplasty with a stiffness for cartilage of around 0.69 MPa,^15^ and autologous chondrocyte implantation with an even lower stiffness, will affect the biomechanics in the surrounding and opposing cartilage, and likely the clinical outcome of the treatment. Another factor known to be vital for the clinical outcome of treatments using osteochondral implants is the placement of the implant. Precise positioning of the implant has been shown to be challenging to achieve consistently.^16^ It is generally accepted that a protruding implant should be avoided and most studies advise placement of the implant flush with, or just below, the adjacent articular surface.^15^, ^16^, ^17^, ^18^, ^19^ Deep placement of the implant can also be damaging.^15^, ^16^, ^18^ From a pilot study performed in another project, it was shown that misalignment of implantation up to 10° is quite common. Koh et al.^20^ showed that if an osteochondral autograft is placed at an angle, the protruding edge should be placed flush with the adjacent surface, such that the lower edge is sunk below the articular surface. However, it is unclear whether there is a certain angulation tolerance, and if such a tolerance is dependent on the mechanical properties of the implant material.

We hypothesize that effects of angulation are smaller for an implant with a low stiffness than for an implant with a high stiffness. This means that a soft implant is expected to be more accommodating for larger deviations from the correct implantation angle than stiffer implants. During loading, cartilage will deform significantly into defects or gaps, resulting in deformations and pressures that are potentially damaging.^21^ When an implant is incorrectly positioned, the area that remains unfilled by the implant is larger compared to when the implant is correctly placed. When an implant with a low stiffness is used, this gap will be compensated for to some extent due to deformations of the implant itself, resulting in a smaller gap. Stiff implants deform less, and thus the gap will be larger. The larger gap resulting from a stiff implant at an angle may cause larger cartilage deformations and thus higher tissue strains. During motion after compressive creep loading, adverse effects from implant angulation and material stiffness are expected to be amplified, because sliding poses additional deformations on the cartilage tissue. In the current study, we aimed to investigate these hypotheses and identify vital design and implantation criteria by employing 3D Finite Element models of cartilage/cartilage contact with a composition‐based material model for articular cartilage. In addition to an intact and defect geometry, implantation angle variations, and implant material stiffness variations were investigated.

## METHODS

### Geometry and Mesh

**Figure 1:**
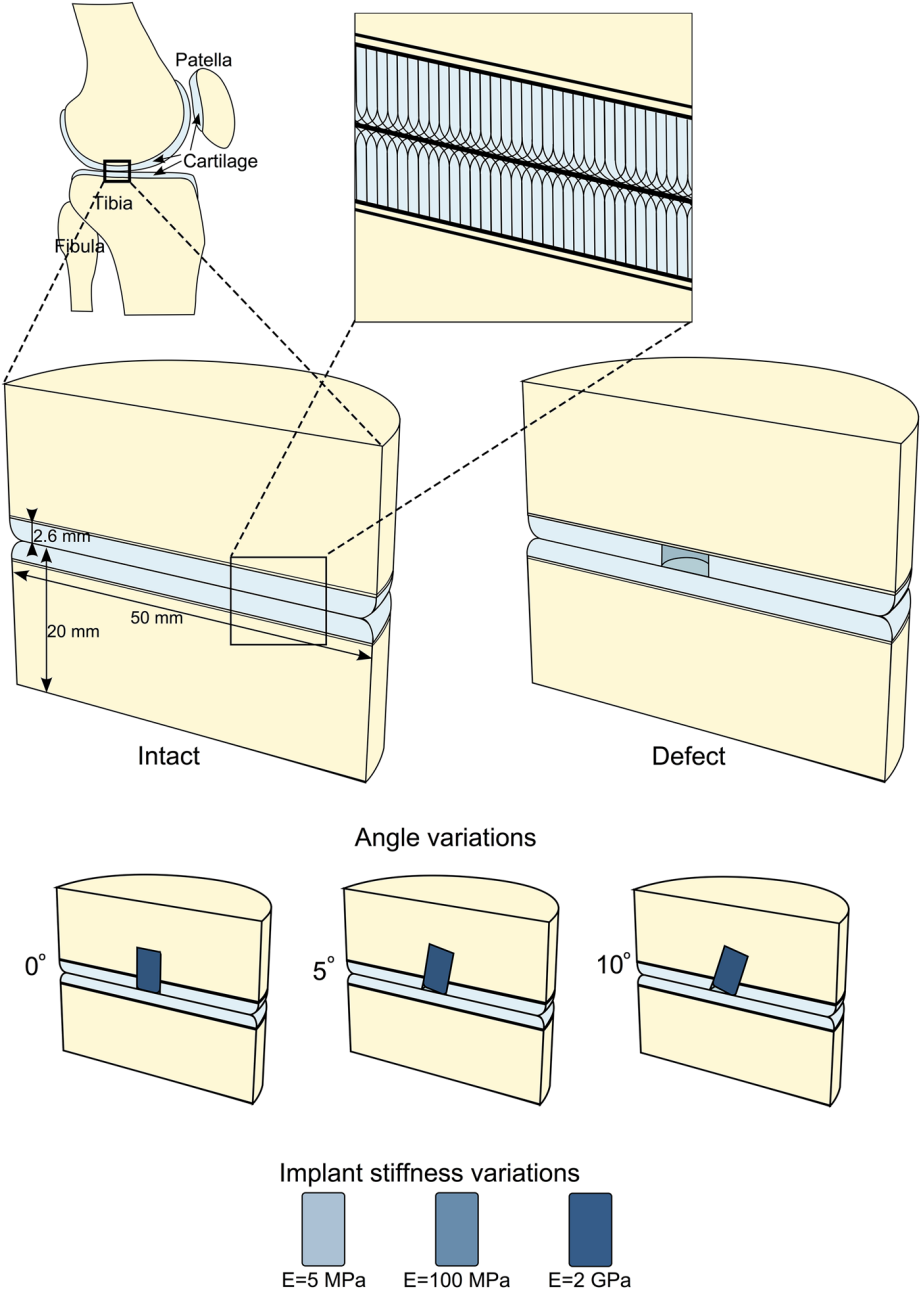
Finite Element geometries for intact and defect (middle row) and implant angle variations (row below). Additional implant stiffness variations are depicted at the bottom. The collagen fiber orientation is dependent on the depth in the articular cartilage (blow up, top).

3D Geometries of simplified cartilage/cartilage contact in the knee joint were developed using Abaqus 2016 (Dassault Systèmes, Vélizy‐Villacoublay, France) for in an intact case, a defect case, and cases in which a defect was treated with an osteochondral implant placed at angles of 0°, 5°, and 10° (Fig. 1). For simplification, menisci were neglected by assuming two flat pieces of cartilage with congruent contact. Due to symmetry, only half of a cylinder was considered. Two parts, with a diameter of 50 mm and thickness of 20 mm, consisted each of a layer of articular cartilage (2.6 mm thick), a subchondral bone plate underneath the cartilage (thickness 0.213 mm), and the rest cancellous bone. The cartilage edges were rounded, to prevent high deformations outside of the area of interest. In the defect model, a circular full‐thickness cartilage defect with a diameter of 6 mm was created at the center of the cylinder. In the implant models of perfect implant placement, this defect was extended into the bone to obtain a defect with a total depth of 10 mm, which was then and filled with a cylindrical implant which fit the space perfectly (diameter 6 mm and height 10 mm). The articular edges of the implant were rounded with a radius of 0.5 mm, which is always done in practice to prevent any sharp edges on the implants. In the implant models with implantation angles of 5° and 10°, the defect was extended a bit more into the bone such that the most prominent edge of the implant was placed flush with the articulating surface with a 10 mm height implant.

Frictionless contact was assumed between cartilage parts. Between implant and cartilage, a coefficient of friction of 0.1 for various biomaterials at time scales around 1 min was assumed.^22^ The contacting surfaces of bone and implants were tied to each other to represent contact without any relative motion, that is, perfect fixation of the implant in the bone.

**Figure 2:**
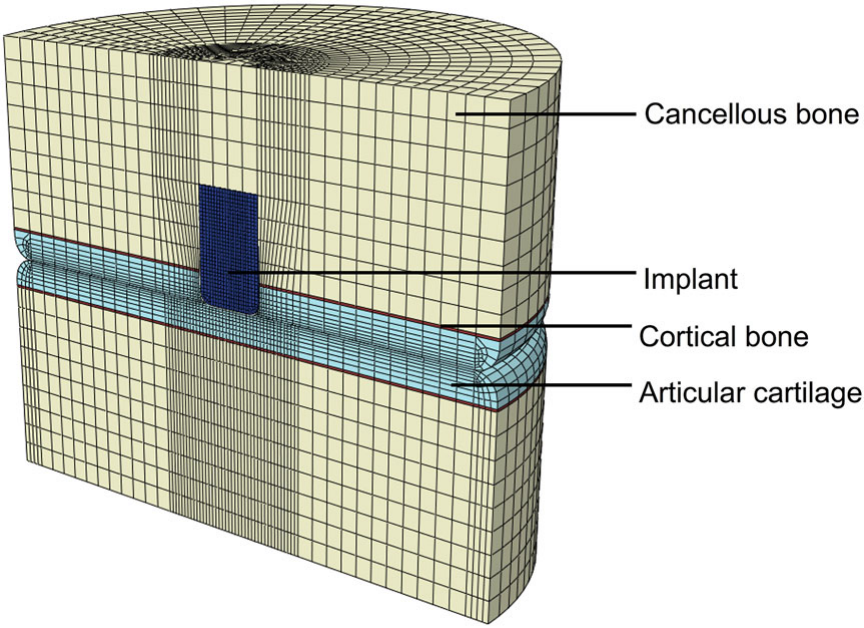
Mesh of the 3D FE geometry for the implant scenario with an implant placed at 0° angulation, with the cancellous bone in yellow, the subchondral bone plate in red, cartilage layers in light blue, and the implant in dark blue.

A total of eight elements covered the thickness of each of the cartilage layers. At the superficial zone, the thickness of elements was 0.176 mm, three times thinner than at the deep zone, to ensure a correct representation of the parallel fibers in the thin superficial zone. Elements at the center of the model were 0.25 mm wide, while at the outer edges, cartilage elements were up to 2 mm wide. This ensured a sufficient number elements in the regions of interest (Fig. 2). The element type used for bone, both cancellous and subchondral, and implant was a linear eight‐node brick reduced integration element (C3D8R). The element type used for cartilage was a linear hexahedral reduced integration pore pressure element (C3D8RP, with enhanced hourglass control).

### Materials

#### Cartilage

A previously developed and validated composition‐based material model for healthy articular cartilage was used to simulate the behavior of cartilage (Fig. 2). This model includes depth‐dependent fiber‐reinforcement, poroviscoelasticity, and swelling behavior.^23^ In the model, the porous solid matrix contains proteoglycans in a non‐fibrillar matrix, and collagen fibers in a fibrillar component of the solid. The collagen fibers form a network, formed by two primary fiber directions in the *x*‐*y* plane per integration point which together form the arcade‐like organization proposed by Benninghof^24^ (Fig. 1, blow up) and seven secondary viscoelastic fiber directions per integration point, representing crosslinks and fibers in random directions. Donan‐Gibbs osmotic swelling is included in the non‐fibrillar matrix, where negatively charged proteoglycans cause a swelling pressure in the tissue which is restricted by the collagen fiber network.^23^

As the model is composition based, each component contributes to the total stress (***σ*** *_tot_*) in the cartilage:

$$\sigma_{tot}=-\mu_{f}I+n_{s,0}\left(\left(1-\sum_{i-1}^{tot\;f}\rho_{c}^{i}\right)\sigma_{nf}+\sum_{i-1}^{tot\;f}\rho_{c}^{i}\sigma_{f}^{i}\right)-\Delta \pi I$$

where *μ_f_* is the fluid pressure (hydrostatic pressure), *n*_*s*,0_ the initial solid volume fraction, $\rho_{c}^{i}$ is the volume fraction of the collagen fibers in the ith direction with respect to the total volume of the solid matrix, ***σ*** *_nf_* is the stress in the non fibrillar part, $\sigma_{f}^{i}$ the stress in the collagen fibers in the ith direction, and Δ*π* is the osmotic swelling pressure. The stress in the non fibrillar network is calculated using a Neo–Hookean model. The stress in the collagen network is given by^25^:

$$\sigma_{f}={\sigma_{}}_{fib}\vec{e_{f}}\vec{e_{f}}+\sigma_{fiso}$$

where $\sigma_{fib}$ is modeled by a spring parallel to another spring in series with a linear dashpot. The springs are modeled using a two‐parameter exponential stress–strain relationship. $\sigma_{fiso}$ is the isotropic stiffness of the fibers, and is described by the same Neo–Hookean model used to describe the stress in the non‐fibrillar network. Both the shear modulus of the matrix and the shear modulus of the collagen fibers are set to 1 MPa.^25^

The density of the primary and secondary fibers are given by^23^:

$$\begin{array}{c} {Primary\;fibers\;\rho }_{c}^{}=\rho_{c,tot}^{}\frac{C}{2C+7}\\ {Secondary\;fibers\;\rho }_{c}^{}=\rho_{c,tot}^{}\frac{C}{2C+7}\\ with\;C=3.0 \end{array}$$

For more details, all other parameter values and for formulations of the osmotic pressure and strain dependent permeability, the reader is referred to the Appendix and Wilson et al.^23^

The model is implemented through a UMAT subroutine in a commercial FE solver (Abaqus 2016, Dassault Systèmes, Vélizy‐Villacoublay, France).

#### Subchondral and Cancellous Bone

Both the subchondral plate and cancellous bone (Fig. 2) are modeled as linear elastic materials with Young's Moduli of 16.16 GPa and 1 GPa, respectively^15^, ^26^ and a Poisson's ration of 0.3.

#### Implants

The implant (Fig. 1) is modeled using a linear elastic material. Three different Young's moduli are used; 5 MPa, 100 MPa, and 2 GPa, with a Poisson's ratio of 0.4. This wide range of Young's moduli corresponds to a wide range of possible materials for osteochondral resurfacing implants, from autographs to soft and harder polymers and even metals.

### Boundary Conditions and Loading

Prior to any loading steps, the cartilage was allowed to equilibrate during a swelling step of 100,000 s in which the external salt concentration was lowered from 2 M to 0.15 M. This was done to reach appropriate initial conditions that mimic the swollen in vivo state of cartilage. The bone surface of the lower part was fixed in all directions and the bone surface of the top part was allowed to move in the vertical direction. Symmetry boundary conditions were active on the front plane. Free fluid flow at the free outer edges of the cartilage was assumed throughout all steps by prescribing a pore pressure of zero. During the loading step, a load of 0.6 MPa was applied to the top bone surface of the upper part in 10 s, followed by a creep step during which the load was kept constant at 0.6 MPa for 500 s. This corresponds with a person of 60 kg bodyweight during one legged stance, with an assumed contact area of 10 cm^2^ per knee.^27^ During a subsequent sliding step of 1 s, the bottom part of the geometry was moved to the right 5 mm to the right. This represents the motion during gait in vivo, displacements in the knee during gait are around −1 mm to +1 mm antero–posterior and 0 mm to −5 mm medio‐lateral, for a flexion angle range of 0 and 70°.^28^, ^29^

Collagen fiber strain, deviatoric strain in the cartilage matrix, and hydrostatic pressure were evaluated as output parameters. Collagen fiber strains are assumed to be indicative of damage to the fibers.^30^ At each integration point two primary collagen fiber components were present in the *x*‐*y* plane, necessary to accurately represent the arcade architecture of the fiber network. The maximum strain value of these two components, in the direction of the fibers, at each integration point was collected and used for further data analysis. Deviatoric strains are a measure for the shape change of the proteoglycan matrix, which is believed to be predictive of damage to the non‐fibrillar matrix.^30^ Different levels of hydrostatic pressures are associated with chondrocyte activation, de‐activation, or even apoptosis.^31^, ^32^ Hydrostatic pressures were calculated by adding the pore pressure, which is hydrostatic pressure in the fluid, at each element to the trace of the stress in the solid fraction divided by 3, which is the hydrostatic pressure in the solid, for each element.

## RESULTS

### Static Creep Loading

**Figure 3:**
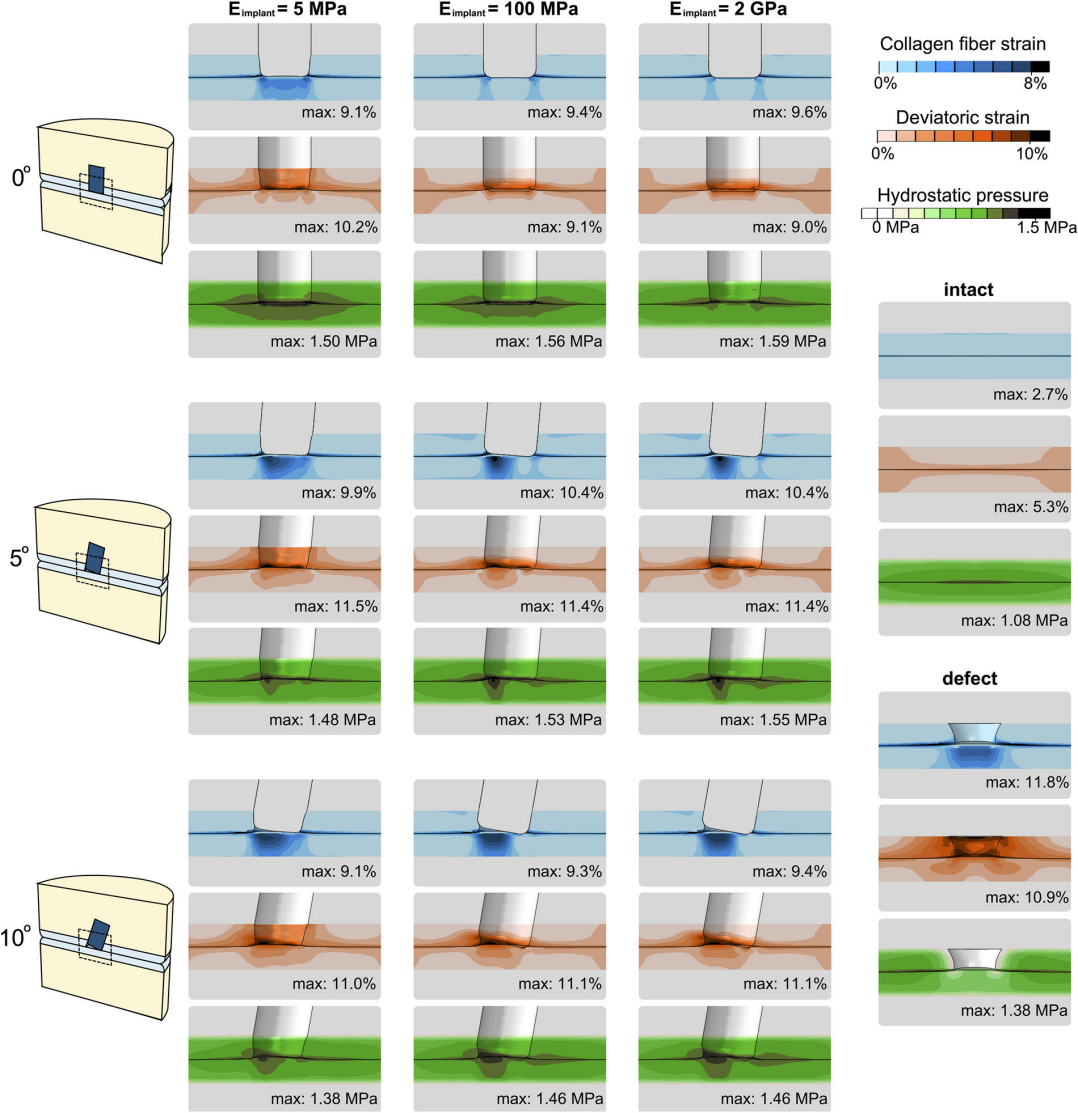
Collagen fiber strains (top of each set), deviatoric strains (middle of each set), and hydrostatic pressures (bottom of each set) resulting from creep loading of 0.6 MPa during 500 s. Strains in cartilage surrounding an implant with a stiffness of 5 MPa are shown in the left column, surrounding an implant with a stiffness of 100 MPa in the middle column and surrounding an implant with a stiffness of 2 GPa are shown in the right column. Top row: Implants at an angle of 0°; middle row: Implants at an angle of 5°; bottom row: Implants at an angle of 10°. Right: Legend with strains for the intact and defect models. The number in the top right corner of each images displays the maximum value in the cartilage for each scenario. To enhance the view for the deviatoric strain and hydrostatic pressure images, the implant was removed from the view.

Collagen fiber strains (maximum 2.7% at the articular surface) and deviatoric strains (maximum 5.3% at the outer edge of the model), were low in the intact model (Fig. 3). Hydrostatic pressures were high in the center and low toward the outer edges of the cartilage where fluid is expelled from the tissue. A defect caused bulging of adjacent and opposing tissue into the defect. This resulted in increased collagen fiber strains in the surface adjacent to the defect (maximum 11.8%) and in the opposing cartilage (maximum 5.4%). At the defect edges, deviatoric strains increased (maximum 10.9%), and hydrostatic pressure decreased. A perfectly placed implant at a 0° angle reduced all above effects, though the extent depended on the stiffness of the implant and the maximum strains remained elevated compared to intact cartilage. Higher implant stiffness resulted in higher collagen fiber strain and hydrostatic pressure maxima, 9.1% and 1.50 MPa for 5 MPa implant compared to 9.4% and 1.56 MPa for the 100 MPa implant and 9.6% and 1.59 MPa for the 2 GPa implant, respectively. Similarly, deviatoric strain maxima decreased with implant stiffness from 10.9.% around an untreated defect, to 10.2% for the 5 MPa implant and 9.1% and 9.0% for 100 MPa and 2 GPa implants, respectively.

When the implant was placed at an angle with one side flush with the surface, the adjacent and opposing tissue bulged into the depressed side when loaded. Collagen fiber strains increased opposing to the gap, deviatoric strains increased adjacent to the gap, and hydrostatic pressures increased opposing to the gap and adjacent to the implant. These effects were more pronounced with increasing angle, and to a lesser extent with increasing implant stiffness.

**Figure 4:**
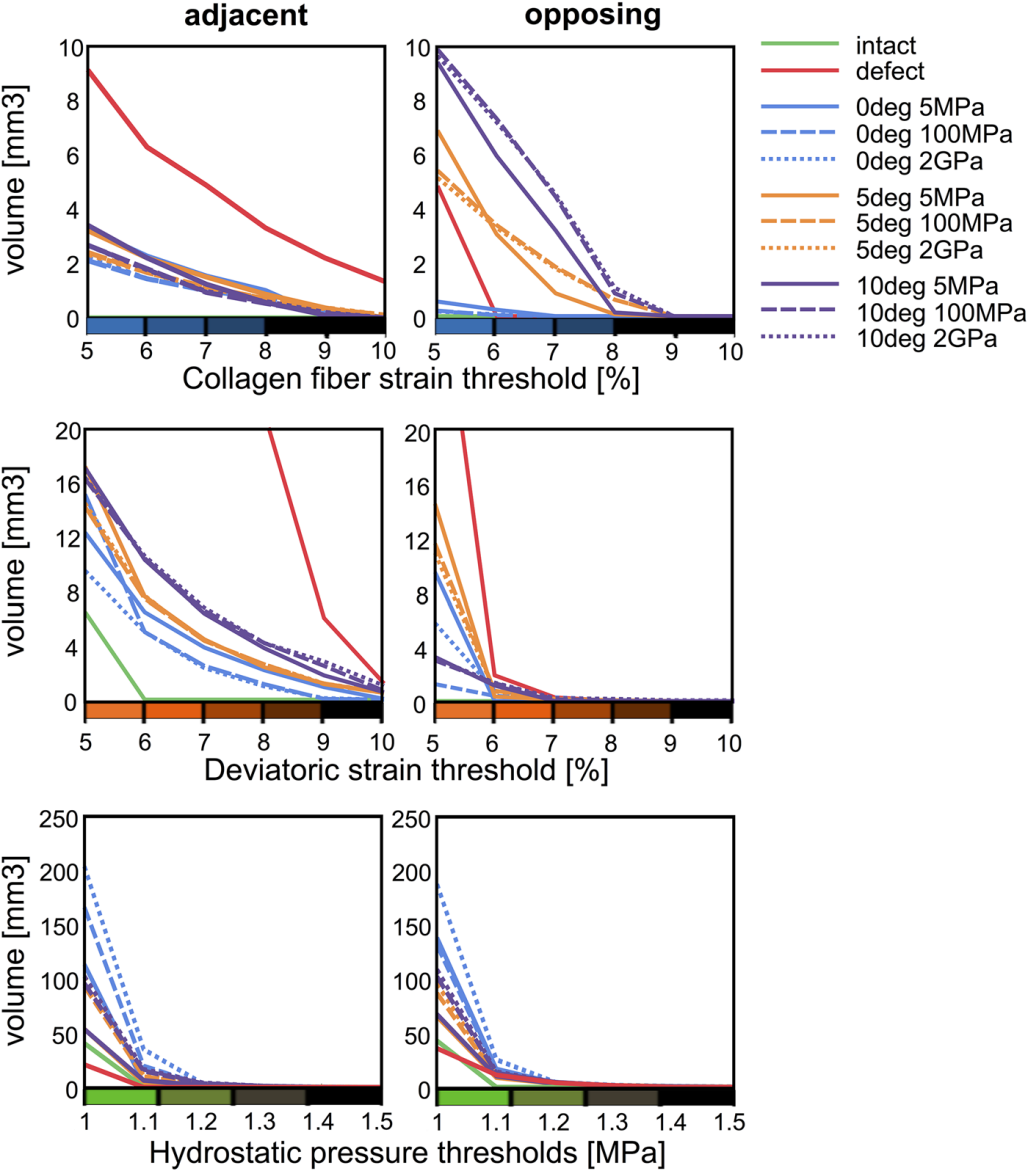
The cartilage volume at which the collagen fiber and deviatoric strains exceed thresholds of 5–10% with intervals of 1% and at which the hydrostatic pressure exceeds thresholds of 1–1.5 MPa, with intervals of 0.1 MPa. Blue lines represent implants at 0°, orange 5°, and purple 10°. Solid lines represent implants of 5 MPa stiffness, dashed 100 MPa and dotted 2 GPa. The green solid line represents the intact scenario and the red line represents the defect scenario. Left: Volume exceeding thresholds in adjacent cartilage, right: Volume exceeding thresholds in the opposing cartilage.

The volume of adjacent cartilage tissue at which collagen fiber strain thresholds are exceeded was always larger for the defect scenario than for any other scenario (Fig. 4). In opposing cartilage, however, the volume exceeding collagen fiber thresholds was larger for implants at an angle than for the defect scenario. Among implants placed under the same angle, the two stiffer implant variations (100 MPa and 2 GPa) were generally associated with larger volumes of high strain than the softest implant. The volume exceeding a particular threshold of deviatoric strain decreased in both opposing and adjacent cartilage after inserting an implant in a defect (Fig. 4). For hydrostatic pressure, implants are associated with larger volumes at which thresholds were exceeded when compared to the defect and intact scenarios.

### Sliding

**Figure 5:**
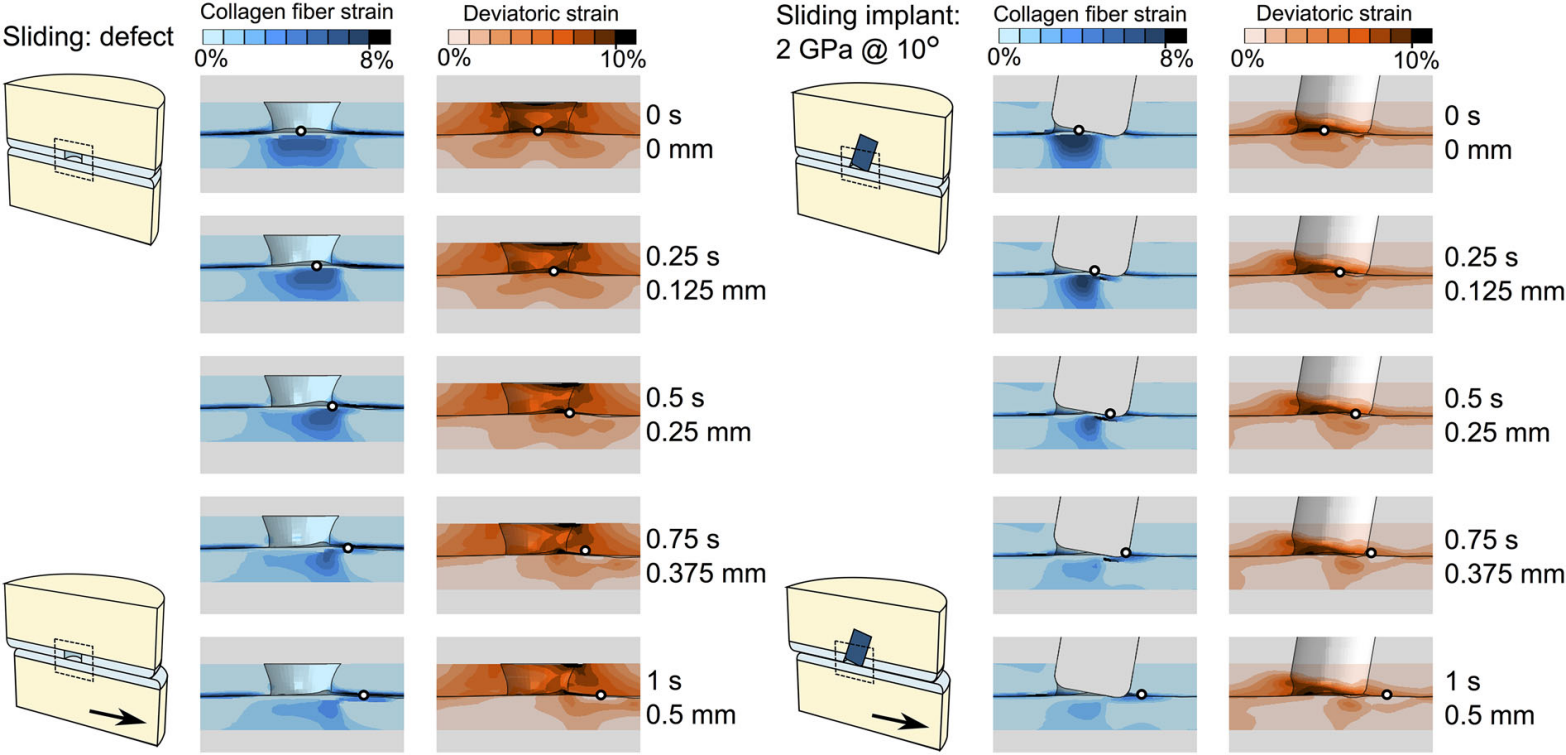
Collagen fiber and deviatoric strain distributions during a 1 s (0.5 mm) sliding step following a 0.6 MPa creep loading for 500 s for the defect scenario (left) and the 2 GPa implant at 10° (right). The small black and white circle indicates the movement of the bottom part to the right, with the top images the start of the sliding step and the bottom images the end of the sliding step. The implant is removed from the view for improved interpretation.

**Figure 6:**
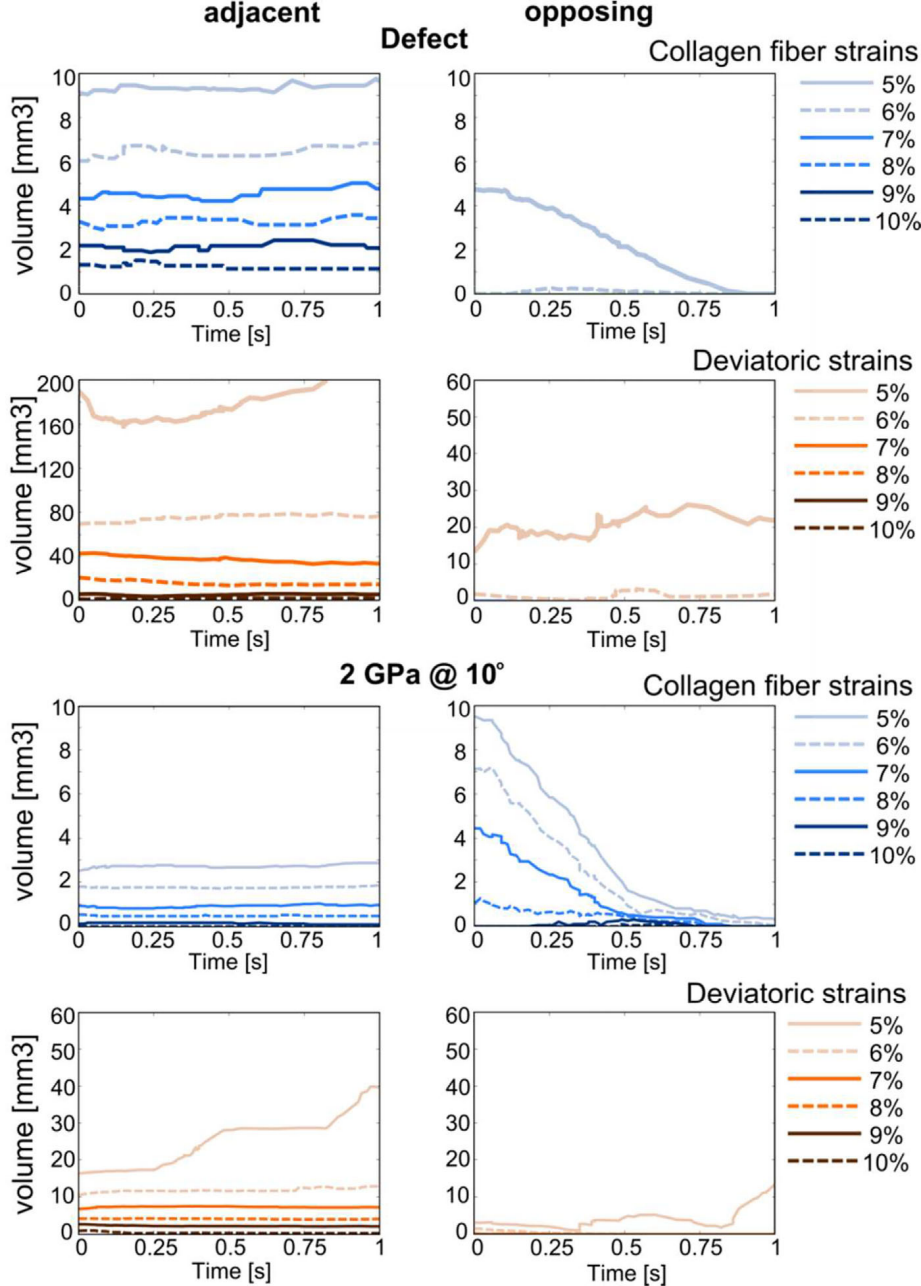
Cartilage volume at which collagen fiber (blue) and deviatoric (orange) strain thresholds are exceeded during a 1 s (0.5 mm) sliding step following a 0.6 MPa creep loading for 500 s. Top four graphs: Defect scenario, bottom four images: 2 GPa implant at 10° angle. Images on the left: Tissue volume adjacent to defect or implant, images on the right: Tissue volume opposing defect or implant.

After equilibration, the opposing cartilage bulged into the defect area, which caused high collagen strains in the superficial zone. Due to the angle that was chosen for the implant, moving the lower cartilage to the left has less impact in terms of friction between implant and cartilage than moving the lower cartilage to the right. Therefore the latter condition was presented in Figure 5. At the onset of sliding, this opposing bulge was pushed against the tissue adjacent to the defect (Fig. 5). Consequently, the bulge decreased in size, and the collagen fiber strains ameliorated. Strains adjacent to the defect remained similar (Figs. 5 and 6). The distribution of deviatoric strains changed, but the tissue volume exceeding thresholds remained relatively constant throughout the sliding step. Collagen strains adjacent to a stiff implant (*E* = 2 GPa) with a 10° angulation also remained similar during sliding, while the collagen fiber strains in the opposing tissue decreased significantly as the local tissue bulge was pushed down. Tissue volume in the adjacent cartilage exceeding a deviatoric strain of 5% increased during the sliding step, but the volume exceeding 6% remained unchanged (Fig. 5). In the opposing cartilage, the volume exceeding strain thresholds remained constant during the sliding step.

One of the simulations, with implant stiffness 2 GPa and implantation angle of 10°, was also done with zero friction between implant and cartilage. The largest difference in maximum collagen fiber strain during the sliding step occurred at the end of sliding with 9.1% strain for the original case compared to 9.4% strain for the frictionless case.

## DISCUSSION

This study focused on the effects of the mechanical stiffness of osteochondral implants combined with the effects of inaccurate implantation angles using 3D Finite Element models of idealized cartilage/cartilage contact in the knee joint. The FEA results demonstrated that focal cartilage lesions caused adjacent cartilage to bulge sideways into the defect, increasing deviatoric strains at the defect edge and tangential fiber strains in the adjacent superficial zone. Low hydrostatic pressures at the defect edges were the result of free fluid flow in this region. Interestingly, the effect of an implant was different for the adjacent and the opposing cartilage. In the adjacent tissue, the mechanical situation was restored toward the healthy state because an implant prevented bulging of surrounding cartilage. This effect was nearly regardless of implant stiffness or angle. In the opposing tissue, however, the mechanical situation depended on implant stiffness and angle. At a perfect implantation angle, stiffer implants (2 GPa) prevented more bulging than softer ones (5 MPa), thus they performed slightly better. However, in neither case, potentially damaging strains were reached. Placement of an implant at an angle with the most prominent edge flush to the surface, resulted in a gap at the depressed side (Fig. 3). During a constant load over 500 s, the porous cartilage tissue released fluid and stretched viscoelastic collagen fibers, which caused the cartilage to deform into the gap, similar to the original defect case. Deviatoric strains surrounding the gap, collagen fiber strains in the surface of the opposing cartilage and hydrostatic pressures opposing the gap increased significantly. Locally, the collagen fiber strains even exceeded those in the case of the untreated defect. Only in the extreme cases, softer material was slightly more forgiving than stiffer material. However, the effect of implantation angle always outweighed the effect of implant stiffness. During sliding motion, after a period of sustained static loading, the bulging tissue was pressed back down, resulting in lower collagen fiber strains in opposing tissue, whereas collagen strains in adjacent tissue and deviatoric strains adjacent and opposing, remained constant throughout the sliding step.

At the rim of the defect, superficial collagen fiber strains increased significantly compared to the intact situation, similar to previous numerical models.^21^, ^33^ This is also where rim stress concentrations were reported experimentally.^34^, ^35^ Deeper in the tissue deviatoric strains increased, in agreement with previous studies.^21^, ^33^, ^36^ Kock et al.^37^ reported that contact pressures surrounding defects can be 190% of the contact pressures in the healthy state, dependent on gait phase,^19^ and on defect and implant size,^15^ which could be ameliorated by mosaicplasty^37^ or a metal osteochondral resurfacing implant.^15^ These effects were also evident from the current predictions for collagen fiber strain, deviatoric strain, and hydrostatic pressure. Positioning errors such as proud or recessed placement have been studied in the past using animal studies, cadaveric knees, and finite element simulations,^15^, ^16^, ^18^, ^19^ but much less research has been done on the angulation errors during osteochondral resurfacing. Koh et al.^20^ studied the effect of angle and depth of osteochondral autograft placement on the resulting contact pressure maps. Although the general magnitudes of their pressures agree with the present findings (data not shown), it is not possible to compare the present data with their work in more detail, due to substantial differences in geometry, loading, and output parameters.

There are several studies which consider damage to the articular cartilage.^38^, ^39^, ^40^ In line with these studies, we consider three damage metrics in this study, each focusing on a separate component of cartilage; collagen fiber strain as a metric for damage to the collagen network, deviatoric strain as a metric for nonfibrillar matrix disruption and hydrostatic pressure as a damage metric for chondrocytes. No experimental data are available on collagen type II fiber strain thresholds for damage, but for collagen type I, the fiber strain at which damage initiates is around 7–10%.^41^, ^42^ These are in the same order of magnitude as the strains found in the superficial zone adjacent to a damaged area. For the nonfibrillar matrix, also no true damage threshold is known. However, a damage initiation strain has been estimated round 30% based on fits between computational evaluations and experimental data.^43^ This deviatoric strain damage threshold is never exceeded in the current simulations. Deviatoric strains may also affect chondrocyte death, as there is evidence that chondrocyte death is dependent on cell aspect ratio.^44^ In addition, chondrocytes are known to be affected by hydrostatic pressures. In vitro culturing studies showed stimulatory effects of hydrostatic pressures of 1–5 MPa, while hydrostatic pressures of 10 MPa and higher decreased activity and induced chondrocyte apoptosis.^31^, ^32^ Hydrostatic pressures at the end of the loading step in the present study are within the stimulatory range. In the beginning of the creep step, hydrostatic pressures were up to roughly 0.7 MPa higher, thus never exceeding the stimulatory range. As the true damage thresholds are unknown, we chose to present the data considering a range of thresholds (Fig. 4). Furthermore, it can be assumed that damage thresholds are affected by aging and disease, because these affect articular cartilage biomechanics. With normal aging, AGEs (advanced glycation endproducts) cause an increased cross‐linking of collagen molecules, which leads to a higher stiffness of the cartilage. In addition, increased proteolytic activity due to age and disease cause an increase in collagen degradation.^45^ Because the exact strain thresholds for cartilage damage are unknown and are likely affected by aging and disease, we chose to present ranges of strain thresholds in our data, rather than identifying or selecting one particular threshold for damage.

A defect caused increased collagen fiber strains and decreased hydrostatic pressures in cartilage opposing and adjacent to the defect. Deviatoric strains were increased in adjacent cartilage. A perfectly placed implant generally benefitted the surrounding tissue compared to an untreated defect, though peak collagen fiber strains still reached potentially damaging values at the implant edges. An implant at an angle, however, did not benefit the surrounding tissue in all aspects; it resulted in increased collagen fiber strains and a larger volume of high collagen fiber strains in the opposing cartilage. On the other hand, elevated deviatoric strains at the defect edges were resolved to some extent by any implant at any angle. These findings confirm that correct placement of an osteochondral resurfacing implant is vital, but they also suggest that over all, implants may have mostly positive effects, regardless of stiffness and angle. Interestingly and contra‐intuitively, rather than causing additional damage, this study reveals how joint motion eliminates high strains which developed in opposing cartilage during prolonged loading. The direction of sliding was chosen to maximize any effect of friction in the contact between implant and opposing cartilage during sliding. In adjacent tissue, collagen fiber strains and deviatoric strains were not affected significantly, neither negatively or positively. Thus, the risk of progression of existing cartilage damage appears to be reduced by regular motion of the joint. Effects of loading magnitude or rotation velocity during this first movement were outside the scope of this study. However, based on the present results, it may be postulated that unloaded or mildly loaded knee flexion after a prolonged period of static loading may be beneficial to cartilage. This also suggests that damage resulting from dynamic loading may result from a different mechanism. Such mechanism could be related to friction‐induced wear and abrasion between implant and cartilage, which can be a focus for future studies.

The Finite Element geometry and meshes were fully 3D, but they were simplified in terms of geometry compared to the human knee joint. The designed geometry omitted ligaments and was completely congruent, representing a perfect fit of the tibia to the femur, which in a real knee joint is the function of the menisci. An interesting next step would be to include incongruence, larger defect sizes, and other knee components. Although the effects of including these features will likely not significantly change the findings of this study, it may amplify them, as the contact area and loading magnitude may be changed and thus local stresses and strains may reach threshold values faster. The implants considered in this study were cylindrical with uniform linear elastic material behavior throughout the whole implant. More advanced designs of the implant, such as using layers with different material properties,^13^ might alter the biomechanical effects on surrounding tissue. However, given that stresses and strains in the cartilage are similar between implants with stiffness ranging from 5 MPa to 2 GPa, it may be expected that the success of such multi‐layered implants also depends more on angulation than on layered material properties. In the current study, a perfect fixation of the implant in the bone was assumed by using a tie constraint. The use of a tighter constraint, resembling a press fit of the implant into the bone, may have an effect on the surrounding bone, but likely not on the cartilage because the misalignment gap will not be changed. However, if the constraint is relaxed, resembling a loose implant, the misalignment gap may become larger, which will increase the effects found in this paper.

## CONCLUSIONS

Based on FEA results from the current study it can be concluded that implants, regardless of correct alignment and material stiffness, have a mostly positive effect on the mechanical conditions in the cartilage adjacent to the implant. Such mechanical conditions include collagen fiber strains, deviatoric strains, and hydrostatic pressure. These effects are superior if the implant is aligned correctly. Opposing cartilage also benefits from an implant, but only when placed at a correct angle. Placement of an osteochondral resurfacing implant at an angle leaves a gap, which causes the opposing cartilage to bulge into the void area at the depressed side of the implant under sustained loading conditions. Consequently, high collagen fiber strains develop in the surface of the opposing cartilage. Therefore, it is concluded that correct placement of implants is crucial for the clinical success of the treatment. Finally, simulations predict that joint motion has beneficial effects on the strain levels in the cartilage surrounding and opposing a full defect or an implant.

## AUTHORS’ CONTRIBUTIONS

All authors, AH, WW, KI, CCvD declare to have had significant contribution to (i) to research design, or the acquisition, analysis or interpretation of data; (2) drafting the paper or revising it critically; (3) approval of the submitted and final versions.

## Figures and Tables

**Figure 7:**
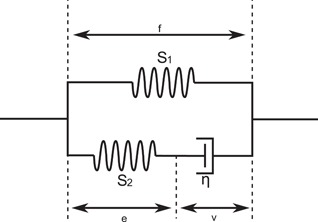
Schematic model for a viscoelastic collagen fibril (*ϵ_f_* is the total fibril strain, *ϵ_v_* the dashpot strain, and *ϵ_e_* the strain in spring *S*_2_).

## Appendix

Complete Model and Parameters

Model

Articular cartilage is assumed as biphasic, consisting of a porous solid matrix saturated with water. The porous solid matrix consists of a swelling nonfibrillar part which contains mainly proteoglycans, and a fibrillary part representing the collagen network. The total tissue stress is given by

$$\sigma_{tot}=-\mu_{f}I+n_{s,0}\left(\left(1-\overset{totf}{\underset{i=1}{\sum }}\rho_{c}^{i}\right)\sigma_{nf}+\overset{totf}{\underset{i=1}{\sum }}\rho_{c}^{i}\sigma_{f}^{i}\right)-\Delta \pi I$$

where *μ_f_* is the water chemical potential, **I** the unit tensor, Δ*π* the osmotic pressure gradient, *n*_*s*,0_ the initial solid volume (in the loaded and nonswollen state), $\sigma_{nf}$ the stress in the nonfibrillar matrix, $\sigma_{f}^{i}$ the fibril stress in the *i*th fibril with respect to the global coordinate system, *totf* the total number of fibril compartments included, and $\rho_{c}^{i}$ the volume fraction of the collagen fibrils in the *i*th direction with respect to the total solid volume.

Fibrillar Part

The fibril stress tensor is given by

$$\sigma_{f}=\frac{\lambda }{J}P_{1}\vec{e_{1}}\vec{e_{1}}$$

where *J* is the determinant of the deformation tensor **F**, *λ* the elongation of the fibril, *P*_1_ the first Piola‐Kirchhoff fibril stress, and $\vec{e_{1}}$ the current fibril direction. The viscoelastic behavior of the collagen fibrils was represented by spring *S*_1_, parallel to spring *S*_2_ in series with a linear dashpot with dashpot constant *η* (Fig. 7).

The mechanical behaviors of springs *S*_1_ and *S*_2_ was determined by two‐parameter exponential stress–strain relationships

$$\begin{array}{cc} P_{i}=E_{i}\left(e^{k_{i}\epsilon_{i}}-1\right) & For\;\epsilon_{i}>0\\ {\begin{array}{c} P \end{array}}_{i}=0 & For\;\epsilon_{i}\leq 0\\ P_{f}=P_{1}+P_{2} & \; \end{array}$$

For *i* = 1,2, and with *ϵ*_1_ = *ϵ_f_* and *ϵ*_2_ = *ϵ_e_*

*E*_1_, *E*_2_, *k*_1_, and *k*_2_ are positive material constants, *ϵ_f_* is the total fibril strain, and *ϵ_e_* is the strain in spring *S*_2_. The total fibril stress is the sum of *P*_1_ and *P*_2­_.

The stresses in the dashpot and the spring *S*_2_ in Figure 1 must be the same. Hence, *P*_2_ can also be given by

$$P_{2}=\eta {\dot{\epsilon }}_{v}=\eta \left({\dot{\epsilon }}_{f}-{\dot{\epsilon }}_{e}\right)$$

where *ϵ_v_* is the dashpot strain, and *η* the dashpot constant.

The fibril structure was implemented as two primary and seven secondary fibril directions. The density of each fibril with respect to the total collagen density is given by

$$\rho_{c}=\rho_{c,tot}\frac{C}{2C+7}\;for\;the\;primary\;\;fibrils$$

$$\rho_{c}=\rho_{c,tot}\frac{1}{2C+7}\;for\;the\;\,\sec ondary\;fibrils$$

With *C* a positive constant greater than 1 and *ρ_c,tot_* the total collagen fiber density. The primary fibers follow the arcade organization as discussed in the methods section, and the seven secondary fibers are dispersed uniformly in space.

The stress in the nonfibrillar solid matrix is given by the following modified Neo–Hookean law:

$$\sigma_{nf}=-\frac{1}{6}\frac{\log\left(J\right)}{J}G_{m}I\left[-1+\frac{3\left(J+n_{s,0}\right)}{\left(-J+n_{s,0}\right)}+\frac{3\log\left(J\right)Jn_{s,0}}{{\left(-J+n_{s,0}\right)}^{2}}\right]+\frac{G_{m}}{J}\left(F\cdot F^{T}-J^{\frac{2}{3}}I\right)$$

where *G_m_* is the shear modulus.

The osmotic pressure Δ*π* gradient is given by

$$\Delta \pi =\phi_{int}RT\sqrt{c_{F,ext}^{2}+4\frac{{\left(\gamma_{ext}^{\pm }\right)}^{2}}{{\left(\gamma_{int}^{\pm }\right)}^{2}}c_{ext}^{2}}-2\phi_{ext}RTc_{ext}$$

$$c_{F,ext}=\frac{n_{f}c_{F}}{n_{ext}}$$

With *n_f_* the total fluid fraction, *n_ext_* the extra‐fibrillar fluid fraction, *C_F_* the normal fixed charge density in mEq per ml total fluid, *ϕ_α_* the osmotic coefficient, γ_α_ the activity coefficients. The external salt concentration (*c_ext_*) was 0.15 M, the temperature (*T*) 293 K, and the gas constant (*R*) 8.3145 × 10^−3^ N m/mmol K.

The permeability is assumed to be strain‐dependent, and given by

$$k=k_{0}{\left(\frac{1+c_{ref}}{1+n_{ext}}\right)}^{M}=\alpha {\left(1-n_{ext}\right)}^{-M}$$

where *k*_0_ is the initial permeability, *c_ref_* a reference value that is constant over the depth of the tissue, *n_exf_* the current extra‐fibrillar fluid fraction, *α* a positive material constant and *M* another positive constant.

The fluid fraction, collagen fraction and fixed charge density distributions were defined as a function of the normalized depth *z**.

The model was implemented in ABAQUS 2016.

**Table:**

Parameter	Value
Gm_pg_, shear modulus of the PG matrix	1 MPa
Gm_coll_, shear modulus of the collagen matrix	1 MPa
*E*_1_, material constant (elastic fibrilpart)	4.3 MPa
*k*_1_, Material constant (elastic fibrilpart)	17 [−]
*E*_2,_ Material constant (visoelastic fibrilpart)	20 MPa
*k*_2_, Material constant (viscoelastic fibrilpart)	41 [−]
*η*, Dashpot of viscoelastic fibrilpart	142400 MPa s
*Α*, Permeability constant	0.00018 mm^4^/(N s)
*M*, nonlinearity term of permeability	1.3 [−]

## References

[1] Hjelle K , Solheim E , Strand T , et al. 2002 Articular cartilage defects in 1,000 knee arthroscopies. Arthrosc J Arthrosc Relat Surg 18:730–734. 10.1053/jars.2002.32839 12209430

[2] Widuchowski W , Widuchowski J , Trzaska T . 2007 Articular cartilage defects: study of 25,124 knee arthroscopies. Knee 14:177–182. 17428666 10.1016/j.knee.2007.02.001

[3] Heir S , Nerhus TK , Rotterud JH , et al. 2010 Focal cartilage defects in the knee impair quality of life as much as severe osteoarthritis: a comparison of knee injury and osteoarthritis outcome score in 4 patient categories scheduled for knee surgery. Am J Sports Med 38:231–237. 20042546 10.1177/0363546509352157

[4] Solheim E , Magnus A , Peder K , et al. 2016 Symptoms and function in patients with articular cartilage lesions in 1,000 knee arthroscopies. Knee Surg Sport Traumatol Arthrosc 24:1610–1616. 10.1007/s00167-014-3472-9 25502829

[5] Buckwalter JA , Mankin HJ , Grodzinsky AJ . 2005 Articular cartilage and osteoarthritis. AAOS Instr Lect 54:465–480. 15952258

[6] Jackson DW , Lalor PA , Aberman HM , et al. 2001 Spontaneous repair of full‐thickness defects of articular cartilage in a goat model. J Bone Jt Surg 83‐A:53–64. 10.2106/00004623-200101000-00008 11205859

[7] Messner K , Gillquist J . 1996 Cartilage repair a critical review. Acta Orthop Scand 67:523–529. 8948264 10.3109/17453679608996682

[8] Takeda H , Nakagawa T , Nakamura K , et al. 2011 Prevention and management of knee osteoarthritis and knee cartilage injury in sports. Br J Sports Med 45:304 LP‐309. 21357577 10.1136/bjsm.2010.082321

[9] Jeuken RM , Roth AK , Peters RJRW , et al. 2016 Polymers in cartilage defect repair of the knee: current status and future prospects. Polymers (Basel) 8:1–30. 10.3390/polym8060219 PMC6432241 30979313

[10] Imhoff AB , Feucht MJ . 2015 Prospective evaluation of anatomic patellofemoral inlay resurfacing: clinical, radiographic, and sports‐related results after 24 months. Knee Surg Sport Traumatol Arthrosc 23:1299–1307. 10.1007/s00167-013-2786-3 24310926

[11] Martinez‐carranza N , Ryd L , Hultenby K , et al. 2016 Treatment of full thickness focal cartilage lesions with a metallic resurfacing implant in a sheep animal model, 1 year evaluation. Osteoarthritis Cartilage 24:484–493. 26403063 10.1016/j.joca.2015.09.009

[12] Nathwani D , McNicholas M , Hart A , et al. 2017 Partial resurfacing of the knee with the BioPoly implant—interim report at 2 years. J Bone Jt Surg 2:1–8. 10.2106/JBJS.OA.16.00011 PMC6132472 30229214

[13] Cook JL , Kuroki K , Bozynski CC , et al. 2014 Evaluation of synthetic osteochondral implants. J Knee Surg 27:295–302. 24281985 10.1055/s-0033-1361951

[14] Messner K , Gillquist J . 1993 Synthetic implants for the repair of osteochondral defects of the medial femoral condyle: a biomechanical and histological evaluation in the rabbit knee. Biomaterials 15:513–521. 10.1016/0142-9612(93)90240-3 8329524

[15] Manda K , Ryd L , Eriksson A . 2011 Finite element simulations of a focal knee resurfacing implant applied to localized cartilage defects in a sheep model. J Biomech 44:794–801. 21300358 10.1016/j.jbiomech.2010.12.026

[16] Martinez‐carranza N , Berg HE , Hultenby K , et al. 2013 Focal knee resurfacing and effects of surgical precision on opposing cartilage. A pilot study on 12 sheep. Osteoarthritis Cartilage 21:739–745. 23428602 10.1016/j.joca.2013.02.004

[17] Kirker‐head CA , Van Sickle DC , Ek SW , et al. 2006 Safety of, and biological and functional response to, a novel metallic implant for the management of focal full‐thickness cartilage defects: preliminary assessment in an animal model out to 1 year. J Orthop Res 24:1095–1108. 16609973 10.1002/jor.20120

[18] Custers RJH , Dhert WJ , van Rijen MH , et al. 2007 Articular damage caused by metal plugs in a rabbit model for treatment of localized cartilage defects. Osteoarthritis Cartilage 15:937–945. 17376710 10.1016/j.joca.2007.02.007

[19] Becher C , Huber ÆR , Thermann H , et al. 2008 Effects of a contoured articular prosthetic device on tibiofemoral peak contact pressure: a biomechanical study. Knee Surg Sport Traumatol Arthrosc 16:56–63. 10.1007/s00167-007-0416-7 PMC2190783 17934718

[20] Koh JL , Kowalski A , Lautenschlager E . 2006 The effect of angled osteochondral a biomechanical study. Am J Sports Med 34:116–119. 16282582 10.1177/0363546505281236

[21] Heuijerjans A , Wilson W , Ito K , et al. 2017 Clinical biomechanics the critical size of focal articular cartilage defects is associated with strains in the collagen fibers. Clin Biomech 50:40–46. 10.1016/j.clinbiomech.2017.09.015 28987870

[22] Chan SMT , Neu CP , Komvopoulos K , et al. 2011 Friction and wear of hemiarthroplasty biomaterials in reciprocating sliding contact with articular cartilage. J Tribol 133:41201.

[23] Wilson W , Huyghe JM , van Donkelaar CC . 2006 A composition‐based cartilage model for the assessment of compositional changes during cartilage damage and adaptation. Osteoarthritis Cartilage 14:554–560. 16476555 10.1016/j.joca.2005.12.006

[24] Benninghoff A . 1925 Form und Bau der Gelenkknorpel in ihren Beziehungen zur Funktion. Z Zellforsch 2:783–862.

[25] Barthelemy VMP , Van Rijsbergen MM , Wilson W , et al. 2016 A computational spinal motion segment model incorporating a matrix composition‐based model of the intervertebral disc. J Mech Behav Biomed Mater 54:194–204. 26469631 10.1016/j.jmbbm.2015.09.028

[26] Ashman RB , Rho JY , Turner CH . 1989 Anatomical moduli variation of orthotropic elastic of the proximal human tibia. J Biomech 22:895–900. 2693453 10.1016/0021-9290(89)90073-0

[27] Fukubayashi T , Kurosawa H . 1980 The contact area and pressure distribution pattern of the knee a study of normal and osteoarthrotic knee joints. Acta Ortho 51:871–879. 10.3109/17453678008990887 6894212

[28] Wilson DR , Connor JJO . 1997 A three‐dimensional geometric model of the knee for the study of joint forces in gait. Gait Posture 5:108–115.

[29] Di Gregorio R , Parenti‐Castelli V . 2003 Pairs for modelling the human. Trans ASME 125:232–237. 10.1115/1.1559895 12751285

[30] Hosseini SM , Wilson W , Ito K , et al. 2014 A numerical model to study mechanically induced initiation and progression of damage in articular cartilage. Osteoarthritis Cartilage 22:95–103. 24185112 10.1016/j.joca.2013.10.010

[31] Elder BD , Ph D , Athanasiou KA , et al. 2009 Hydrostatic pressure in articular cartilage tissue engineering: from chondrocytes to tissue regeneration. Tissue Eng Part B 15:43–53. 10.1089/ten.teb.2008.0435 PMC2817666 19196119

[32] Nakamura S , Arai Y , Takahashi KA , et al. 2006 Hydrostatic pressure induces apoptosis of chondrocytes cultured in alginate beads. J Orthop Res 24:733–739. 16514637 10.1002/jor.20077

[33] Venäläinen MS , Mononen ME , Salo J , et al. 2016 Quantitative evaluation of the mechanical risks caused by focal cartilage defects in the knee. Sci Rep 6:37538. 27897156 10.1038/srep37538 PMC5126640

[34] Papaioannou G , Demetropoulos CK , King YH . 2010 The knee predicting the effects of knee focal articular surface injury with a patient‐specific finite element model. Knee 17:61–68. 19477131 10.1016/j.knee.2009.05.001

[35] Peña E , Calvo B , Martínez MA , et al. 2007 Effect of the size and location of osteochondral defects in degenerative arthritis. A finite element simulation. Comput Biol Med 37:376–387. 16796999 10.1016/j.compbiomed.2006.04.004

[36] Dabiri Y , Li L . 2015 Focal cartilage defect compromises fluid‐pressure dependent load support in the knee joint. Int J Numer Method Biomed Eng. 32 10.1002/cnm.2713. 25727068

[37] Kock NB , Smolders JMH , Susante Van JLC , et al. 2008 A cadaveric analysis of contact stress restoration after osteochondral transplantation of a cylindrical cartilage defect. Knee Surg Sport Traumatol Arthrosc 16:461–468. 10.1007/s00167-008-0494-1 PMC2358931 18292989

[38] Párraga Quiroga JM , Wilson W , Ito K , et al. 2017 The effect of loading rate on the development of early damage in articular cartilage. Biomech Model Mechanobiol 16:263–273. 27514541 10.1007/s10237-016-0815-0 PMC5285418

[39] Mononen ME , Tanska P , Isaksson H , et al. 2018 New algorithm for simulation of proteoglycan loss and collagen degeneration in the knee joint: data from the osteoarthritis initiative. J Orthop Res 36:1673–1683. 29150953 10.1002/jor.23811

[40] Men tao Y , Jiang long Y , Chen L , et al. 2017 On mechanical mechanism of damage evolution in articular cartilage. Mater Sci Eng C 78:79–87. 10.1016/j.msec.2017.03.289 28576051

[41] Zitnay JL , Li Y , Qin Z , et al. 2017 By collagen hybridizing peptides. Nat Commun 8:1–12. 28327610 10.1038/ncomms14913 PMC5364439

[42] Legerlotz K , Riley GP , Screen HRC . 2010 Specimen dimensions influence the measurement of material properties in tendon fascicles. J Biomech 43:2274–2280. 20483410 10.1016/j.jbiomech.2010.04.040 PMC2935962

[43] Wilson W , van Burken C , van Donkelaar C , et al. 2006 Causes of mechanically induced collagen damage in articular cartilage. J Orthop Res 24:220–228. 16435355 10.1002/jor.20027

[44] De Vries SAH , Van Turnhout MC , Oomens CWJ , et al. 2014 Deformation thresholds for chondrocyte death and the protective effect of the pericellular matrix. Tissue Eng Part A 20:1870–1876. 24438476 10.1089/ten.tea.2013.0436 PMC4086379

[45] Lotz M , Loeser RF . 2012 Effects of aging on articular cartilage homeostasis. Bone 51:241–248. 22487298 10.1016/j.bone.2012.03.023 PMC3372644

